# Chronic Discharges from the Ear and Their Treatment

**Published:** 1898-12-03

**Authors:** Macleod Yearsley

**Affiliations:** Assistant-Surgeon to the Royal Ear Hospital; Surgeon-In-Charge of the Department for Diseases of the Throat, Nose, and Ear, the Farringdon General Dispensary; Hon. Surgeon for Diseases of the Throat and Ear, the Governesses's Home, &c.


### HOSPITAL CLINICS AND MEDICAL PROGRESS.

### CHRONIC DISCHARGES FROM THE EAR AND THEIR TREATMENT.

By MACLEOD YEARSLEY, F.R.C.S., Assistant-Surgeon to the Royal Ear Hospital; Surgeon-in-Charge of the Department for Diseases of the Throat, Nose, and Ear, the Farringdon General Dispensary; Hon. Surgeon for Diseases of the Throat and Ear, the Governesses's Home, &c.

(*Read before the South-West London Medical Society, November 9th,* 1898.)

THE importance of chronic purulent inflammation of the middle-ear cannot be over-estimated. Its frequency, the disturbances of nutrition to which it may give rise; and last, but not least, the dangerous character of its complications and sequelæ render it a disease to be dealt in earnest, and not to be combatted by half-hearted attempts at treatment. In no form of inflammation of the middle-ear is the hearing apparatus subject to such extensive changes—changes which may not only do irreparable damage to the hearing power, but may at any moment put the patient's life in imminent danger.

Chronic suppuration in the middle-ear is most frequent in childhood, and is more generally bilateral than confined to one side. The character of the discharge is usually purulent, and it is but rarely that we find more mucus than pus. The amount of discharge is greatest in the scarletino-diphtheritic form, or when there are extensive granulations, or caries of the temporal bone, or in the consecutive formation of abscesses in the region of the ear. The odour of the discharge is of comparatively little diagnostic value, but there are one or two points in regard to it that are of use to us in prognosis and treatment. If the smell speedily disappears under simple antiseptic treatment the prognosis is certainly more favourable, for the persistence of fœtor points usually to the retention of pus in the antrum or to caries of the ossicles or of the temporal bone. When it is clear that the discharge appeared without previous pain, especially when the condition is bilateral, and occurs in a weakly child, suspicion should be entertained of tubercle, and we should satisfy ourselves as far as possible that such is not the case before giving an opinion to the contrary. On examining a case of chronic middle-ear suppuration the first thing to do is to ascertain the nature, quantity, and smell of the discharge, and the presence or absence of pain in or around the ear. The next point is to gently syringe out the ear. When this has been done a careful examination can then be made with a speculum under a good reflected light, and a variety of conditions may be encountered. Putting aside the presence of polypi or granulations, attention should be paid to four points: (1) The locality and size of any perforation of the tympanic membrane; (2) the condition of the rest of the membrane; (3) the condition of the inner wall of the tympanum; (4) the condition of the external auditory meatus.

The most frequently occurring perforation is one situated in the anterior-inferior portion of the membrana tympani. The next is one in the posterior-superior quadrant, and the third most common is one in Shrapnell's membrane. When the perforation is small and the discharge is profuse, the former may be very difficult to see, but a depression in which is pulsating fluid reflecting the light strongly will often indicate its situation, and the diagnosis may be confirmed by other methods than that of inspection. The most useful methods at our disposal for diagnosing a perforation are inspection through a speculum, the use of Siegle's pneumatic speculum, and the production of the perforation sound or whistle. The latter may be obtained in two ways; a diagnostic tube brings the observing ear and the patient's ear into communication, and the tympanum is inflated by means of Politzer's douche or the Eustachian catheter. The sound of the air whistling through the perforation is a distinctive one, and once heard cannot be mistaken. Should there, however, be a difficulty in thus inflating the tympanum on account of some co-existent Eustachian obstruction, the inflation may be performed *viâ* the external meatus, the diagnostic tube being inserted in the patient's nostril.

Perforations in the superior-posterior quadrant of the membrane are often very difficult to treat, because they are so frequently complicated by caries of the incus. Perforations in Shrapnell's membrane often run a very tedious and obstinate course, are very frequently complicated by caries of the head of the malleus, the body of the incus, and of the external attic wall. They are furthermore liable to be followed by suppuration in the mastoid antrum. If a perforation is fairly large the condition of the inner wall of the tympanum may be readily seen. The mucous membrane covering it usually varies in colour from yellowish-red to scarlet, and may present adherent patches of exudate, pus, &c. The tumefaction seen may be trifling or considerable, and, if the latter, may be sometimes mistaken for a polypus. Granulations may also be seen springing from the inner tympanic wall. The principal complications of chronic middle-ear suppuration are granulations, polypi, disease of the ossicles, and temporal bone, mastoid involvement, and cholesteatomata. The sequelæ are meningitis, cerebral or cerebellar abscess, lateral sinus thrombosis, and pyæmia.

Time will not permit me to deal with the sequelæ. The complications, however, have an important bearing upon treatment, and must, therefore, be discussed. Disease of the ossicles has already been mentioned. Involvement of the mastoid antrum may be regarded as a subject to be dealt with separately, but the occurrence of granulations and polypi and of cholesteatomata require attention. The former are circumscribed elevations developed by a partial hyperplasia of the infiltrated mucous membrane lining the tympanic cavity. They take the form of simple granulations or of pedunculated polypi. The latter, growing through a perforation in the membrane, may present quite a slender pedicle, and may mask the perforation by filling it entirely and overlapping its edges. They are seen as projecting masses, usually of a lightish red colour. It is important to remember how easily they are hidden by profuse discharge, so that they may very easily be missed if the ear be not syringed carefully before examination with a speculum. In structure polypi are usually of two forms, small round-celled (identical with granulation tissue), or fibrous tissue; the latter is, of course, only a further development of the former. Myxomatous tissue is rare. The formation of cholesteatomata is due to an excessive desquamation of epithelial cells in the external meatus and mucous membrane of the tympanum. If this desquamation be not too excessive, and forms no hindrance to the exit of pus, it may continue for years without giving any trouble, but when excessive it may lead to the formation of large collections of dead epithelial masses in the tympanic cavity. In such a case the results of the formation of these masses is that they not only form an obstruction to the exit of pus, but they swell by imbibition of moisture (such as after syringing, exposure to steam, or in very damp weather), and may cause acute exacerbations of the chronic middle-ear trouble. The result of a cholesteatomatous formation may be a fatal one, by pyemia following retention of septic material behind the mass, or by meningitis, or some other of the sequelæ already mentioned. Their diagnosis is, therefore, important, not only on account of their danger to the patient, but also because the middle-ear suppuration cannot be made to cease before the mass is removed. The diagnosis can be made with certainty only when the masses of dead epithelium lie in view, but the frequent finding of malodorous gritty masses, or sodden strings in the solution used for syringing the ear should arouse suspicion of their presence. Recurrent pain and inflammatory exacerbations should also be regarded as of significance.

(*To be continued.*)

